# Transcriptional regulation as a dose-dependent process: insights from transcription factor tuning

**DOI:** 10.1098/rsob.240328

**Published:** 2025-08-06

**Authors:** Gemma Noviello

**Affiliations:** ^1^ Epigenetics and Neurobiology Unit, European Molecular Biology Laboratory Rome, Monterotondo, Roma 00015, Italy

**Keywords:** transcription factor, tuning, dose-dependent process, binding sites affinity, CRISPR, degron

## Abstract

Several biological processes, including transcriptional regulation by transcription factors (TFs), are dose-dependent. At the mathematical level, dose-dependent processes can be modelled by fitting dose–response curves, for istance, employing Hill-type equations. At the experimental level, however, quantitatively regulating, or tuning, endogenous gene expression to characterize dose-dependent processes is challenging. Here, existing methods to fine-tune endogenous gene expression are compared and contrasted. Relatively small TF dosage variations have been shown to underpin cell fate decisions. Nonetheless, the current understanding of the molecular mechanisms by which TFs quantitatively regulate gene expression is limited, due to the paucity of genome-wide studies in endogenous and physiological conditions. Recent works combining quantitative perturbations of TFs and genome-wide response analyses are untangling an underexplored layer of transcriptional control. At the same time, new questions are emerging in the field, which will require further technological advancements in order to be addressed.

**Figure:**
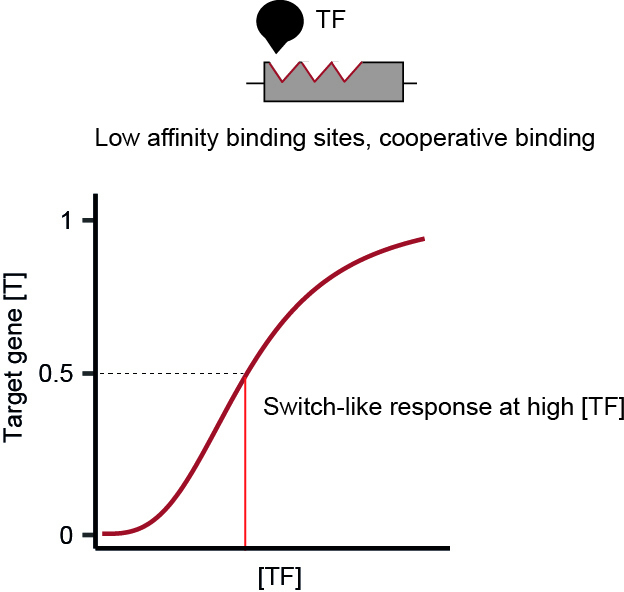

## 1. Dose-dependent processes and their mathematical quantification

**Figure 1.**
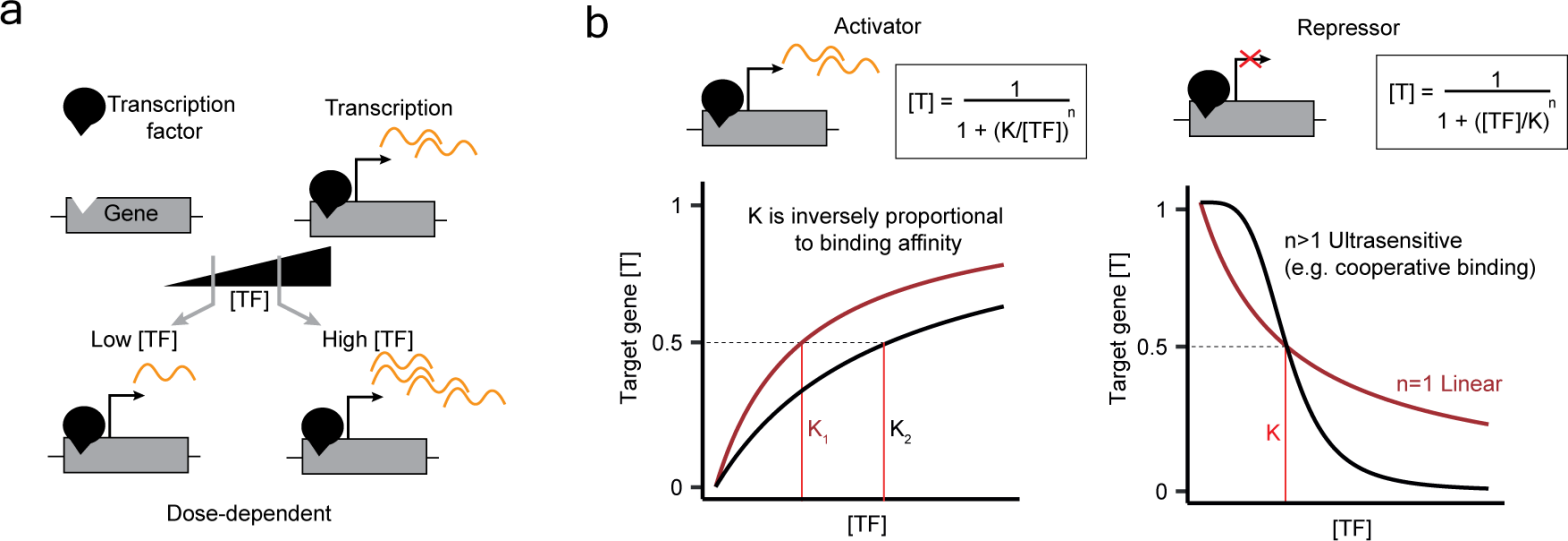
Transcriptional regulation by transcription factors as a dose-dependent process. (a) The concept of dose-dependent processes applied to transcriptional regulation. A transcription factor (TF) binds the promoter of a target gene, leading to its activation. We define gene activation as a dose-dependent process when it varies with the dose of TF, in physiologically relevant ranges. (b) The input function of a gene can be modelled with a Hill curve. The fractional expression of a target gene ([T]) (normalized by the maximal expression) is a function of the quantity of the TF ([TF]). The Hill equation is shown on the left for an activator TF and on the right for a repressor. K is the threshold level of [TF], giving half of the maximal expression of [T]; n is the Hill coefficient. For the activator (left) the two curves are set with n = 1 (linear) and K is changed. For the repressor (right), K is kept constant and n is set to 1 or 3.

Biological processes can be defined ‘dose-dependent’ when they rely on the specific quantity or stoichiometric ratio of their components. A simple example might be the transcription of a gene controlled by a transcription factor (TF). If we vary the quantity of TF within physiologically relevant ranges and observe a variation in the expression of the target gene, the process can be classified as dose-dependent (figure 1a). Naively, one could think that all biological processes are, to some extent, dose-dependent, but this depends on what response we are measuring. As an example, heterozygous mutations of genes can cause a halving of the final correctly transcribed mRNA quantity. Nonetheless, the defect could be compensated by mechanisms such as increased protein translation, decreased protein degradation and redundant factors. Therefore, at a higher scale, the defect might be buffered and, ultimately, undetectable. Indeed, more than 90% of human pathogenic mutations are recessive, but gene-dosage defects are an important cause of dominant diseases, such as haplo-insufficiency (HI) [1,2]. Human HI genes often encode factors involved in transcriptional regulation and are frequently associated with developmental phenotypes [2,3].

A convenient way to study dose-dependent processes is by analysing their dose–response curves, i.e. how the biological response changes when quantities of components are changed. Dose–response curves can be broadly subdivided into linear and nonlinear. Linear dose–responses follow the law of mass action [4] with a Michaelis–Menten type of dynamics, whereby the behaviour is linear at low concentrations and tends to saturation at high concentrations [5]. Nonlinear processes are those not simply proportional to the amounts of their components. A commonly employed equation for modelling dose–response curves is the Hill equation. In particular, it is of interest that the parameters of a Hill curve can have a direct biological meaning in the study of transcriptional regulation. The input function of a gene (i.e. how the expression of a gene changes with the quantity of an activator or repressor TF binding at its promoter) can be modelled with a Hill curve (figure 1b), having two key parameters, K and n [6]. The constant K represents the amount of activator/repressor producing half of the maximal expression of the gene and is inversely related to the affinity of the TF for the target sequence. The Hill coefficient (n) indicates the amount of binding cooperativity. Cooperativity occurs when the binding of a TF to its cognate site increases the affinity of other TFs binding simultaneously [7]. This can occur when multiple molecules of activator/repressor need to co-bind the gene promoter to give rise to a response. Non-cooperative binding will have *n* = 1 (linear dose–response), while cooperative binding will have *n* > 1. Hence, the higher the degree of cooperativity, the more sigmoidal and nonlinear the dose–response curve will be. A classic example of cooperative binding is the activation of the Lac operon by the inducer IPTG: the process requires the co-binding of two IPTG molecules and has been described with a Hill curve having *n* = 2 [8].

Note that the Hill equation is often an over-simplification of the complex relationship between TF concentration and target gene expression. Some assumptions of this equation do not necessarily hold true in real biological systems. For instance, the reactions should happen at an equilibrium state, while cells are spatiotemporally dynamic environments. Moreover, the TF concentration in the equation should represent the concentration available at functional binding sites (BS), which is not equal to the total TF concentration, especially when the TF is lowly expressed and mostly bound at non-functional and/or non-specific BS [9]. Importantly, the equation does not take into account competitive binding of TFs, or the presence of specific cofactors, which will alter the shape of the dose–response curve [7,10,11].

Overall, Hill-type curves have the advantage of parameter interpretability and can fit both linear and nonlinear relationships, but there are cases where other types of equations are necessarily required to describe the dose–response between a TF and a target gene. In complex and interconnected processes, such as TF regulation within transcriptional networks, dose–response curves can, for instance, be non-monotonic [12–14]. This is the case of Nanog’s transcription in response to OCT4 dose variation in mESCs. OCT4 directly activates *Nanog* by binding at its promoter, and when OCT4 is strongly depleted, Nanog transcription also decreases [12,13]. However, when OCT4 dose is roughly halved, Nanog transcription surprisingly is found to increase [15,16].

In summary, dose–response curves are a convenient way to model biological dose-dependent processes, and in particular transcriptional regulation, to better describe the underlying molecular mechanisms. Measuring dose–response curves in living cells and in physiological contexts requires the ability to quantitatively perturb (or tune) endogenous gene expression. In the following section, some methods that can be applied for tuning endogenous gene levels are compared.

## 2. Methods for tuning endogenous gene expression

An ideal approach to study dose-dependent processes is to perturb with high precision the dose of a factor of interest, within physiologically relevant ranges, and measure the resulting response. Endogenous gene expression can be quantitatively regulated at the transcriptional or post-transcriptional level with several techniques, as summarized in table 1.

**Table 1.** Summary of methods to tune endogenous gene expression.

system	tuning mechanism	strengths	weaknesses	references
inducible promoters	Promoter integrated upstream of gene; expression modulated by graded levels of an external inducer (e.g. chemical)	several inducible promoters are available (e.g. Tet-ON/OFF, ERT2, Gal4); potential for orthogonal tuning of multiple genes	requires genetic manipulation; leakiness in uninduced state; intermediate expression levels difficult to achieve; overexpression might not reflect physiological levels	[17–21]
CRISPRi/a	dCas9 fused to activator/repressor domains (e.g. VPR, KRAB)+sgRNA to target promoter region	no need for genetic editing at the gene locus; strong up/down regulation	traditional systems often only allow binary ON/OFF states; overexpression might not reflect physiological levels	[22–25]
degron-dCas9-hHDAC4(CasTuner)	dCas9 fused with hHDAC4 and degron domain; ligand titration controls dCas9-hHDAC4 stability and repression level	no need for genetic editing at the gene locus; shown to allow expression fine-tuning with single-cell resolution	some heterogeneity in repression might be observed. Not tested at the genome-wide level	[16,25]
RNAi (siRNA)	siRNAs bind to mRNA of target gene, triggering its degradation via the endogenous RNAi pathway. siRNAs with varying activity provide intermediate expression of target gene	no need for genetic editing at the gene locus; widely available tool for many species; potential for partial knockdown using different siRNAs	presence of important off-target effects; requires the use of a siRNA for each expression level to achieve	[26–30]
degron-CasRx (CasTuner)	Cas13d (CasRx) is a CRISPR system targeting and degrading mRNA. Ligand titration controls CasRx stability and repression level	no need for genetic editing at the gene locus; shown to allow expression fine-tuning with single-cell resolution	some heterogeneity in repression might be observed. Not tested at the genome-wide level	[16]
CasRx using guides with varying activity	CasRx repression can be tuned by guide activity; sgRNAs with varying activity provide intermediate expression of target gene	no need for genetic editing at the gene locus; shown to allow expression fine-tuning with single-cell resolution	requires the use of a sgRNA for each expression level to achieve; functional validation in single-cell context still limited	[31]
degrons fused to protein of interest (POI)	degron tags added to protein; ligand or condition controls degradation, altering protein abundance post-translationally	rapid kinetics; maintains endogenous expression regulation	requires genetic editing; basal degradation even without ligand can be present; potential structural/functional impact; may not work for all proteins	[32,33]
inducible localization tags fused to POI	protein localization controlled by tags (e.g. light-sensitive); switches between active/inactive compartments (e.g. cytoplasm ↔ nucleus)	fast induction (especially with light tags); spatial control	limited to proteins with compartment-specific activity; shares degron-like drawbacks (e.g. folding/interference)	[34–37]

Transcriptional regulation can be achieved with methods such as inducible promoters or epigenetic editing systems. An inducible promoter can be integrated at the 5′ end of the gene of interest [17,18], and graded levels of inducer (e.g. a chemical) are used to establish intermediate expression levels. However, inducible promoters can be difficult to tune, as they can present basal expression in the uninduced state (leakiness) [19,20] and intermediate expression levels can be difficult to achieve [21], while high expression levels (overexpression) may not reflect the physiological function. Other tools that can transcriptionally regulate gene dosage, but do not require genetic engineering at the locus, are CRISPR-based interference and activation systems (CRISPRi/a) [22–24]. Those systems involve the catalytically dead form of Cas9, dCas9, able to bind a gene of interest via a single guide RNA (sgRNA), fused with effector domains that can regulate target gene transcription, such as transient activators (e.g. VPR) or repressors (e.g. KRAB). Typically, CRISPRi/a systems are used to strongly down- or upregulate target genes. Further technological advancements allowed the exploitation of CRISPRi systems to fine-tune gene expression (reviewed in [25]). To this extent, the fusion of a conditional destabilizing domain (degron) to a dCas9-hHDAC4 (human histone deacetylase 4) proved to be a powerful system[16]. In this system, named CasTuner, titrated concentrations of a ligand are used to quantitatively control the amount of degron-dCas9-hHDAC4 in the cells and, in turn, the amount of repression of a target gene. Of note, when the KRAB domain, a commonly used repressor in CRISPRi systems, was swapped with hHDAC4, gene expression could not be tuned homogeneously across cells but could only be switched OFF completely, indicating that tuning by epigenetic editing relies on specific repression mechanisms. Several degron-based CRISPRa systems have also been developed [25], but their ability to quantitatively control gene expression has not been yet analysed at the single-cell level yet.

Cellular concentration of a protein can also be tuned at the post-transcriptional or post-translational level. In RNA interference (RNAi), short interfering RNAs (siRNA) complementary to an mRNA of interest can be used to knock-down its expression via the endogenous RNAi machinery [26]. It has been shown that different siRNAs targeting the same transcript can induce different knock-down levels [27,28], providing a potential mechanism to quantitatively perturb gene expression. The main flaw of RNAi when used for gene tuning is the limited specificity of the siRNA, which can contribute to important off-target effects [29,30]. Recently, the RNA-targeting Cas9 ortholog RfxCas13d (CasRx) was shown to allow fine-tuning of gene expression levels. This could be achieved either by controlling CasRx abundance using a degron system (as for the dCas9-hHDAC4 system described above) [16], or by employing sgRNAs with intermediate activity [31].

Degron domains themselves can be fused at the 5′ or 3′ of the coding sequence of the gene of interest to tune its expression at protein level [32]. Degrons offer several advantages compared to other tuning systems: they provide fast induction kinetics, and they do not bypass but rather act on top of the endogenous regulation of a gene [33]. However, some major drawbacks are the basal (uninduced) degradation of the protein of interest, possible impediments in structural folding and interactions of the fusion protein. Additionally, the function of these domains on all proteins is not guaranteed. An alternative to degrons are inducible localization tags, which enable regulation of the localization of a protein between the cytoplasm and the nucleus. As such, they could be applied for protein tuning only when a protein is active in a single compartment (e.g. a TF acting within the nucleus). They can theoretically present the same issues as degrons, although some light-inducible localization tags present faster kinetics compared to degrons [34–37].

Advancements in gene tuning technologies might streamline the discovery and characterization of dose-dependent biological processes. A specific field of application is the study of transcriptional regulation by TFs, whereby gene tuning could help both in precisely controlling cell fate decisions, and in understanding the very basic rules of gene regulation. In the next two sections, I first give examples of cell fate trajectories controlled by TF dosage, and then describe some of the key new findings on how TF dose controls gene expression at the molecular level.

## 3. Dose-dependent processes shape development

During development, genes need to be expressed in the correct spatiotemporal frame and, additionally, their level of expression needs to be quantitatively controlled to direct cell decisions. Multiple morphogen gradients are integrated in a concentration-dependent manner to specify the body axes and tissues of an organism [38]. These and other input signals are typically connected to the cell’s transcriptional response (output) via signal transduction cascades that regulate the activity of TFs. How TF inputs are decoded at the genetic level in a quantitative fashion is unclear, but that such a process is crucial for the cell’s ability to maintain its identity or commit to specific lineages is supported by a range of examples. In mouse embryonic stem cells (mESCs), there is a small group of factors named ‘core pluripotency factors’ that are central to the transcriptional regulation that stabilizes the pluripotent state: Sox2, Oct4/Pou5f1 (hereby referred as Oct4) and Nanog. For each of them, dose-dependent effects have been described, both in the maintenance of the pluripotent state, and in the differentiation onto specific lineages, in mouse and human [39,40]. Interestingly, for SOX2 and OCT4, it has been shown that relatively small variations in their levels can drive completely different cell fates. OCT4 upregulation by less than twofold causes mESCs to express markers of the primitive endoderm and mesendoderm lineages, whereas reduced levels initiate the trophectoderm transcriptional programme [41,42]. A roughly twofold increase in SOX2 levels causes loss of pluripotency and expression of a range of differentiation-related genes [43]. These observations are particularly relevant, considering that Oct4 and Sox2 are used for transgene-mediated reprogramming of adult somatic cells into induced pluripotent stem cells (iPSCs) [44,45]. Indeed, efficient reprogramming into iPSCs requires the expression of these factors within specific stoichiometric levels [46,47].

The same concerns should be taken into account when considering other reprogramming and differentiation approaches, which typically rely on the expression of specific TFs [48]. In fact, TF dosage also dictates more specific functions at later stages of development. As an example, specific doses of SOX2 are required for the proper function of retinal cells in the eye and the differentiation of lingual progenitors into taste cells [49,50]. Therefore, understanding the dosage-dependent effects of TFs is key both to studying their context-dependent function and to harnessing their potential to (re)program cells for therapeutic purposes.

## 4. Learn-by-tuning: how transcription factor dose controls the transcriptional output

TFs bind their genomic sites (BS) to regulate transcription, and are critical to the establishment of expression profiles underpinning cell identity and developmental programmes. Sequences preferentially bound by a TF are known as motifs [51]. TF motifs are relatively short (6–25 base pairs) and can occur millions of times in the genome, with only a fraction bound by a TF [51,52]. Furthermore, structurally similar TFs within the same family can drive distinct transcriptional programmes despite sharing a preference for the same motifs. The ability of TFs to specifically recognize their BS to control transcription, among millions of possible genomic sites, has been encapsulated in what is known as the ‘specificity paradox’ [53]: how can TFs find their specific sites among millions of possible ones? Both DNA accessibility and chromatin context influence the binding probability of a TF at a genomic site [54,55]. Chromatin accessibility is a major determinant of TF binding [56], with the majority of TFs binding at open chromatin regions. Some TFs have been termed ‘pioneer’ based on the ability to bind target motifs at inaccessible regions and increase chromatin accessibility [57,58]. A recent study underscored how well-characterized pioneer TFs, Zelda and Grainy head, but also the non-pioneer TF Twist, show concentration-dependent binding and chromatin opening of closed sites when expressed at different concentrations in *Drosophila melanogaster* S2 cells [59]. This work suggests that, in principle, many other TFs could display pioneer properties, when expressed at sufficiently high levels, while also underlining the relevance of studying TF-mediated gene regulation at biologically meaningful concentrations.

Chromatin context, including chromatin structure, histone marks, DNA methylation, the DNA sequence surrounding the motif, presence of other BSs for antagonistic and cooperative TFs, and availability of cofactors, have all been reported to play a role in determining TF binding [55,60–64]. TF concentration interplays with the chromatin context in determining DNA binding and the downstream transcriptional response.

TFs can bind BSs with varying affinity. Because low-affinity BSs are, by definition, less frequently bound, they are also difficult to detect by genome-wide occupancy profiling techniques (e.g. ChIP-Seq, CUTnRun), making it difficult to disentangle their biological relevance [65]. In well-characterized *cis*-regulatory regions, such as the *ZRS* mouse enhancer, it has been shown that redundant low-affinity BSs are important for correct limb development, and a small increase in affinity of one of these sites (from 0.15 to around 0.25) causes formation of extra digits (polydactyly) [66]. This is in line with evidence that low-affinity BSs are crucial for gene regulation and development [67].

**Figure 2.**
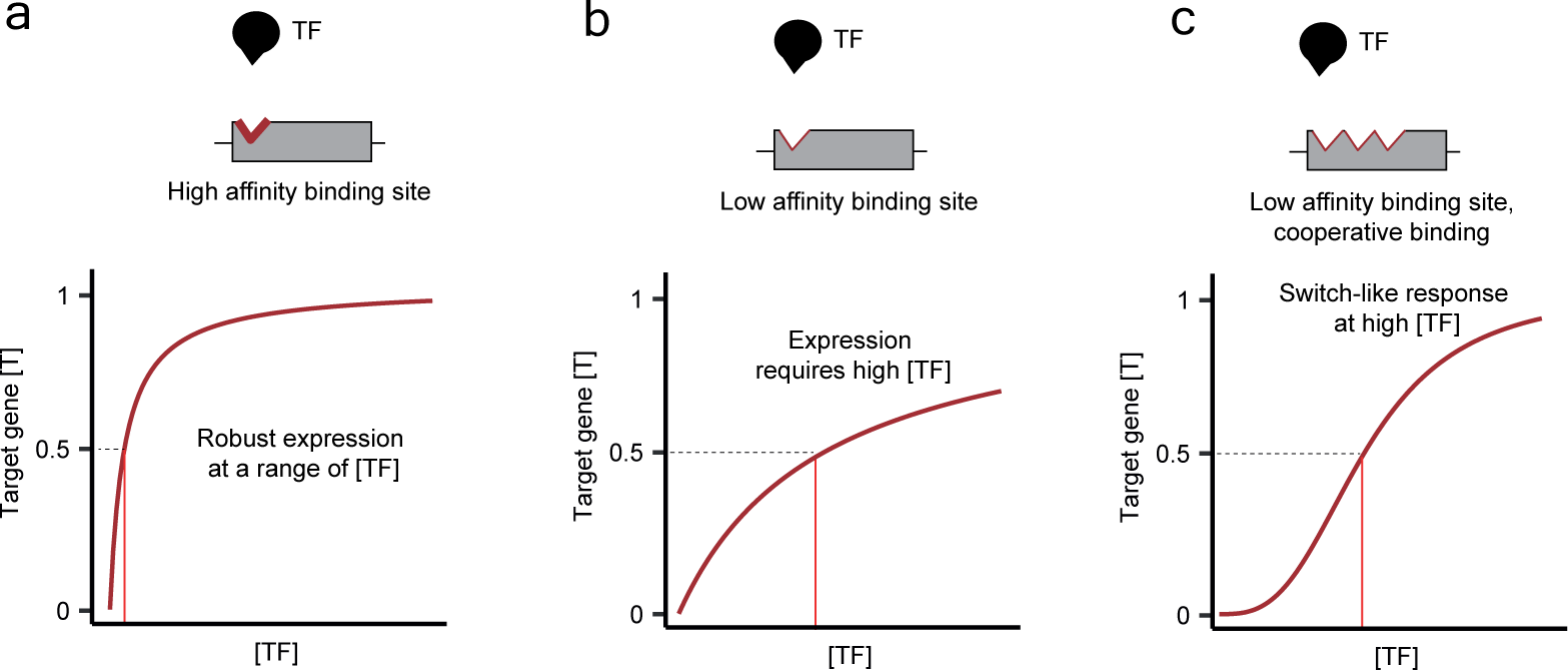
Effects of binding site affinity and composition on dose–response curves. (a) Hill curve for a target gene having a single-binding site (*n* = 1) with high affinity for a TF (small K). (b) Hill curve for a target gene having a single-binding site with low affinity for a TF (K one order of magnitude higher than for (a)). (c) Hill curve for a target gene having three low-affinity binding sites for a TF. Here, n is set to 3, to simulate a case of cooperative binding, while K is the same as in (b).

In a simplified view, given a BS with a specified affinity for a TF, TF concentration will dictate if the site will be occupied (as the binding process is dynamic, it will influence the binding frequency and dwell time [68]). High-affinity BSs tend to remain occupied even at lower TF concentrations and can, for instance, ensure robustness to variations in TF abundance (figure 2a). Low-affinity motifs tend to be bound only at high TF concentrations and can grant tissue- and TF paralogue-specificity [53,69] (figure 2b). BSs can occur in clusters, further modifying the response to TF dosage. Moreover, binding of TFs to clustered sites can occur cooperatively or non-cooperatively (figure 2c). For example, the TF NF-kB was shown to activate its target genes in human cancer cells in a dose-dependent manner, reflecting the amount of pro-inflammatory stimulus to which the cells were exposed. NF-kB could activate some of its key targets in a graded manner by non-cooperative binding to clustered high-affinity BSs. This was suggested to allow tuning of the level of inflammatory response in proportion to the external inflammatory stimulus [70]. Clusters of low-affinity BSs can also mediate transcriptional activation within physiologically relevant TF concentrations. For example, a cluster of three weak BSs could gain similar TF occupancy as a single motif with affinity one order of magnitude higher and drive similar expression levels in *Saccharomyces cerevisiae* [71].

Research on the dose-dependent control of transcription by TFs has mostly been undertaken *in vitro*, using exogenous expression systems or focusing on few transcriptional targets. Recently, pioneering work from Naqvi *et al.* enabled the study of the dose-dependent control of transcription by an endogenously expressed TF at the genome-wide level [32]. The authors engineered a degron system at the *SOX9* locus to tune the endogenous SOX9 protein level. *SOX9* is a HI gene and a master regulator of chondrogenesis. Mild SOX9 dosage variations (approximately within 75% of wild-type) are associated with phenotypic diversity in humans, while heterozygous mutations cause a spectrum of developmental diseases, including Pierre–Robin sequence [32,72]. Combining genome-wide analysis of transcriptional response, TFs binding and chromatin accessibility allowed the authors to explain part of the characteristics of the dose–response curves for target genes. Within SOX9 direct target BSs, they could identify sites that were more sensitive to SOX9 dosage (i.e. with higher K when fitted to an Hill equation, see figure 1b), which were enriched for the SOX9 palindrome motif, and sites that were more buffered to SOX9 variation, which correlated with open chromatin and binding by other TFs. Importantly, genes associated with more dose-sensitive BS, responded more sensitively to SOX9 dose variation, drawing a causative link between dose-dependent TF binding and transcriptional response. The dose-dependent regulation of target genes by SOX9 could also partly explain the association of SOX9 dosage disbalance with diseases: genes that responded sensitively to SOX9 intermediate depletion were associated with Pierre–Robin sequence phenotypes, while genes more robust to SOX9 titration were associated with severe dominant craniofacial abnormalities causing lethality. This study is of particular significance as it connects TF dosage to transcriptional responses in a genome-wide manner and, importantly, to phenotypic responses *in vivo*. Recently the authors applied the same perturbation strategy to another dose-dependent TF involved in craniofacial development, TWIST1 [73]. By training a deep-learning model on data obtained from graded perturbations of TWIST1, as well as of SOX9, they could identify sequence features (syntax) affecting BS sensitivity to dosage of the two TFs. Interestingly, for TWIST1 but not for SOX9, low-affinity BSs were predictive of TF dosage-sensitive binding. This work suggests that there are sequences within the *cis*-regulatory code that might fine-tune the transcriptional response to TF dosage, and that those so far largely unexplored syntax rules might be TF-specific.

## 5. Open questions

In this article, I have discussed the concept of dose-dependent process, along with a simple mathematical model (the Hill equation) that can be useful in quantifying sensitivity and nonlinearity of a dose–response curve. Experimentally, dose–response curves can be obtained by tuning the level of a factor of interest and measuring the associated response. Several methods can allow tuning of endogenous gene expression, although each has specific advantages and disadvantages. I then focused on TFs and reported examples of relatively mild variations of TF dosage that can direct cell fate decisions. At the molecular level, TFs coordinate transcriptional programmes by quantitatively interacting with *cis*-regulatory sequences embedded in various chromatin contexts. Because of a lack of suitable quantitative perturbation strategies in physiological and endogenous contexts, relatively little was known about how TFs control their targets in a dose-dependent manner. Recent studies have paved the way to gain a molecular understanding of these processes. The following are open questions that I hope will stimulate discussions and will be tackled in the next years.

### 5.1. What are the most suitable methods to achieve precise quantitative perturbation?

Gene expression can be, and often is, noisy [74], meaning that for a given gene there can be large variations in expression between theoretically identical cells. Heterogeneity in gene expression can have functional roles or simply be a byproduct of the mechanisms of gene expression itself [74,75]. Nonetheless, when studying dose-dependent processes and, in particular nonlinear ones, it is important to achieve precise control over gene quantity as relatively small variations can drive completely different responses, complicating the analysis when measuring the involved biological entities at a bulk level. To address this challenge, single-cell RNA-sequencing (scRNA-seq) has been employed to measure the dose–response relationship between level of TF and target gene expression [14,76]. Theoretically, scRNA-seq should provide accurate genome-wide dose–response measurements independently of how precise the TF perturbation is. However, not only are scRNA-seq techniques prohibitive in cost for many research laboratories [77], but they are also inaccurate at detecting lowly expressed genes [78], although there are variants allowing measurement with accuracy panels of several hundred genes [79,80]. Given these challenges, it would be important to benchmark different tuning methods on their ability to uniformly control intermediate expression states, in particular for CRISPR-based systems, which potentially allow the tuning of any gene in a less laborious way compared to genetic engineering approaches (e.g. knock-in of degrons).

### 5.2. What are the most suitable methods to fine-tune gene upregulation?

There are cases in which quantitative upregulation of gene expression is desirable. On the one hand, gene overexpression can cause cytotoxicity and non-physiological effects [81]. On the other hand, precise upregulation of gene levels can underline cell fate transitions and improve (re)programming protocols, as discussed before. CRISPRa systems could potentially be repurposed for tuning gene upregulation, as it has been done for CRISPRi, but it remains to be verified whether specific activation mechanisms can work in a gradual manner, homogeneously across cells, or they rather act digitally, by switching ON gene expression in a fraction of cells.

### 5.3. What are the dose-dependent effects of epigenetic marks?

As discussed before, TFs quantitatively control target genes in different dose-dependent manners. Chromatin marks can also instruct transcriptional states at target genes [82]. There are indications that different chromatin marks quantitatively control gene expression with different modes. For example, H3K9me3 has been associated with switching genes OFF completely, while histone acetylation with gradual transcriptional activation [16,83]. By precisely tuning the level of chromatin modifications at loci of interest (e.g. by regulating the domain breadth of the deposited mark), the dose-dependent modes of action of chromatin marks could be resolved, also potentially improving the ability to predict transcriptional states from epigenomic profiles.

### 5.4. Are new methods required to precisely control the local concentration of transcription factors?

In this review, I have discussed approaches and case studies where the total quantity of factors of interest is quantitatively controlled across cells. A strong assumption of these studies is that the tuned factor is homogeneously distributed in the cell or nucleus. In reality, this is not the case, as several membrane or membrane-less compartments exist in eukaryotic cells [84]. Transcriptional processes are compartmentalized in nuclear hubs where the concentration of TFs and cofactors can be dramatically different from the rest of the nucleus [53], influencing the probability of binding and activating target genes. Indeed, the ability to manipulate local concentration of a TF revealed that transcription is under the influence of the local environment [85]. Hence, an additional layer of regulation, i.e. transcription occurring in a heterogeneous three-dimensional environment, modulates gene expression in response to TF dosage. General methods to fine-tune local protein concentration might thus enable to dissect the relative importance of TF concentration local variation.

### 5.5. Is biology moving from being quantifiable to being quantitative?

The ability to systematically quantify all the components present in a cell, which greatly expanded thanks to single-cell ‘omic’ techniques, is enabling to depict with striking accuracy what exactly a cell is and what distinguishes one cell from another [86]. However, we still need perturbations to decipher whether certain differences are functional or stochastic, for example. In other words, the ability to quantify biological molecules at increasingly fine scales does not inherently translate into a mechanistic and quantitative understanding of cellular processes. To this extent, emerging methods for fine-tuning gene expression are proving indispensable to draw a causal nexus between quantity of biological entities and biological effect. Quantitative perturbations, in combination with genome-wide transcriptomic and epigenomic profiling, alongside *ad hoc* computational approaches [32,73,87] are setting the stage for predicting the full spectrum of quantitative expression variation, from molecular mechanisms to phenotypic consequences. Excitingly, these studies have thus far been applied to only a few genes or specific contexts, leaving much yet to be discovered.

## Data Availability

This article has no additional data.
